# Approaches to long-read sequencing in a clinical setting to improve diagnostic rate

**DOI:** 10.1038/s41598-022-20113-x

**Published:** 2022-10-09

**Authors:** Erica Sanford Kobayashi, Serge Batalov, Aaron M. Wenger, Christine Lambert, Harsharan Dhillon, Richard J. Hall, Primo Baybayan, Yan Ding, Seema Rego, Kristen Wigby, Jennifer Friedman, Charlotte Hobbs, Matthew N. Bainbridge

**Affiliations:** 1 Rady Institute for Genomic Medicine, San Diego, CA USA; 2 Department of Pediatrics, Cedars-Sinai Medical Center, Los Angeles, CA USA; 3 Pacific Biosciences, Menlo Park, CA USA; 4 Department of Pediatrics, University of California San Diego and Rady Children’s Hospital, San Diego, CA USA; 5 Department of Neuroscience, University of California San Diego and Rady Children’s Hospital, San Diego, CA USA

**Keywords:** Medical genomics, Medical genetics, Clinical genetics, Paediatrics

## Abstract

Over the past decade, advances in genetic testing, particularly the advent of next-generation sequencing, have led to a paradigm shift in the diagnosis of molecular diseases and disorders. Despite our present collective ability to interrogate more than 90% of the human genome, portions of the genome have eluded us, resulting in stagnation of diagnostic yield with existing methodologies. Here we show how application of a new technology, long-read sequencing, has the potential to improve molecular diagnostic rates. Whole genome sequencing by long reads was able to cover 98% of next-generation sequencing dead zones, which are areas of the genome that are not interpretable by conventional industry-standard short-read sequencing. Through the ability of long-read sequencing to unambiguously call variants in these regions, we discovered an immunodeficiency due to a variant in *IKBKG* in a subject who had previously received a negative genome sequencing result. Additionally, we demonstrate the ability of long-read sequencing to detect small variants on par with short-read sequencing, its superior performance in identifying structural variants, and thirdly, its capacity to determine genomic methylation defects in native DNA. Though the latter technical abilities have been demonstrated, we demonstrate the clinical application of this technology to successfully identify multiple types of variants using a single test.

## Introduction

Despite improvements in sequencing technology, phenotyping, analysis techniques, and collective underlying understanding of the morbid genome, clinical diagnostic rates of genetic disorders have remained relatively static over the past decade^1–3^. The significant step from exome sequencing to short-read whole genome sequencing (SRS) enabled identification of copy number and structural variants, as well as non-exonic splicing and regulatory variants. Though each consecutive methodologic advancement has incrementally improved diagnostic yield, the majority of sequenced patients still remain undiagnosed^4,5^.

Long read sequencing (LRS) has several advantages over SRS^6–8^. Long reads are two orders of magnitude greater in length than short reads, for which the DNA fragments average only a few hundred base pairs in size. Thus, long reads have the ability to map into repetitive or duplicated regions of the genome (e.g. ALUs and pseudogenes) that short reads cannot, due to the inherent ambiguity of which portion of the genome was the source of the short sequenced DNA fragment. These low-complexity regions have been termed next-generation sequencing (NGS) dead zones^9^. In addition, short reads may struggle with particular structural variants that are mediated by repetitive elements and repeat expansions, although a multitude of tools exist to try and alleviate these issues^10–13^. Single-molecule long-read sequencing has the additional advantage of directly detecting epigenetic markers, typically methylated CpG dinucleotides, which can potentially be diagnostic for a number of diseases. The ability of LRS to deduce the methylation profile by sequencing native DNA is another advantage of LRS that traditional SRS is not capable of achieving^14^.

Herein we examine the increase in diagnostic rate from applying LRS to 30 probands with severe, predominantly syndromic pediatric disease phenotypes who had previously received negative genomic results with SRS. Prior analysis for these negative SRS genomes was done by American College of Medical Genetics (ACMG)-boarded laboratory directors and included utilization of established bioinformatic tools to detect structural variants, cryptic splicing variants, mobile insertion elements, and repeat expansions. Lastly, we evaluate the ability of short reads to identify variants in NGS dead zones by force calling variants in these regions.

## Results

### Positive control samples

A total of 35 samples (30 subjects from 26 families to 5 controls; Table 1 and Supplemental Table 1) were sequenced from whole blood using HiFi long-read sequencing. Genome coverage ranged from 25.2 to 38.8× based on available banked DNA, with four outlier samples at 8.5–18.1× because DNA was depleted by initial clinical tests.

**Table 1:** Five controls with previously reported diagnoses.

ID	Variant	Rationale for selection as a control
C1	der (22), t (11;22)	Complex chromosomal rearrangement
C2	t (9;18) (q33;q21.2)	Translocation unable to be validated by Sanger sequencing
C3	Inv (8q13.3; 8q24.22)	Diagnostic inversion
C4	*IKBKG*: p.E222*fs*	NGS dead zone
C5	UPD Chr15	Imprinting/methylation defect

**Figure 1:**
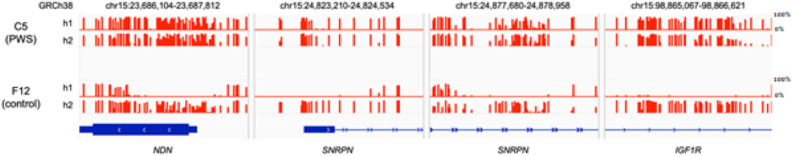
Biallelic hypermethylation on chr15 in Prader–Willi syndrome (PWS). Methylation analysis of HiFi reads shows hypermethylation of both haplotypes at known chr15 imprinted loci in a male patient, C5, with Prader–Willi Syndrome. An unrelated, unaffected male control, F12, shows hypomethylation of one allele. HiFi reads are phased by sequence into haplotype 1s and 2. Values show the percent of reads from each haplotype that are methylated at each genomic CpG site. *h1* haplotype 1, *h2* haplotype 2.

Five previously diagnosed samples were used as controls (Table 1): (C1) a child with Emanuel syndrome (derivative supernumerary chromosome 22 and 11 fusion; MIM: 609029)^15^; (C2) an apparent translocation between chromosomes 9 and 18; (C3) a diagnostic inversion that disrupts *EYA* [MIM:602588]; (C4) a two base deletion in *IKBKG* [MIM:300636]^16^ (VCV000429392); (C5) uniparental heterodisomy of chromosome 15 causing Prader–Willi syndrome [MIM:176270]. LRS was conclusive for all five controls. For cases C1, C3 and C4, the causal variant was correctly identified by LRS (Supplementary Fig. 1). For case C5, methylation analysis showed biallelic hypermethylation at known maternally imprinted loci on chromosome 15, including exons of *MAGEL2*, *NDN*, and *SNRPN*. (Fig. 1). For case C2, LRS indicated that the apparent translocation was instead an insertion of a processed pseudogene, *SMAD4*, into the intron of *SCAI* (Supplementary Fig. 1).

Next, the ability of LRS to replicate small variants identified by SRS across the genomes of 32 samples was evaluated. Overall, LRS robustly reproduced the SRS results, with 99.6% of SNVs and 96.9% small insertions or deletions correctly identified (Supplementary Table 2). Of the remaining SNVs, 0.8% were detected by LRS only, compared to 0.4% of SNVs found only by SRS. The remainder of the small insertions/deletions was close to evenly split between the two, with ~ 2% of variants being unique to each sequencing modality.

### Cases

We selected 30 cases that had received negative diagnoses by SRS. All cases were previously analyzed by our standard clinical pipeline^17^. In brief, DRAGEN alignment and small variant calling was used, followed by CNV and SV variant calling by a consensus of read-depth and read-pair-based methods (minimalistic Parliament), and in-house developed population frequency filtering and prioritization workflows^17^. Subsequently, the SRS data were also analyzed by our plan-Beta pipeline, which interrogates the genome for structural variants using Tiddit^18^, Delly^19^, and GridSS^20^, and repeat expansions using GangSTR and Expansion Hunter, microCNVs (in house developed), mobile insertion elements (in house developed), cryptic splicing mutations using Splice AI and scap, and 5ʹ UTR start gains (in house developed)^11,18–22^. The majority of these cases (n = 22) were identified by nominations from pediatric subspecialists in the divisions of Clinical Genetics, Neurology, Critical Care, and Metabolics, who felt that the patient’s diagnosis was likely genetic in origin despite a negative SRS genomic result. The majority of these patients were syndromic and severely affected. Four more case were affected family members of the 22 clinician-nominated cases. In addition, we selected four patients with a specific phenotype (immune deficiency), for a total of 30 cases (Supplementary Table 1).

### Determination of phenotypes most likely to benefit from LRS

**Figure 2:**
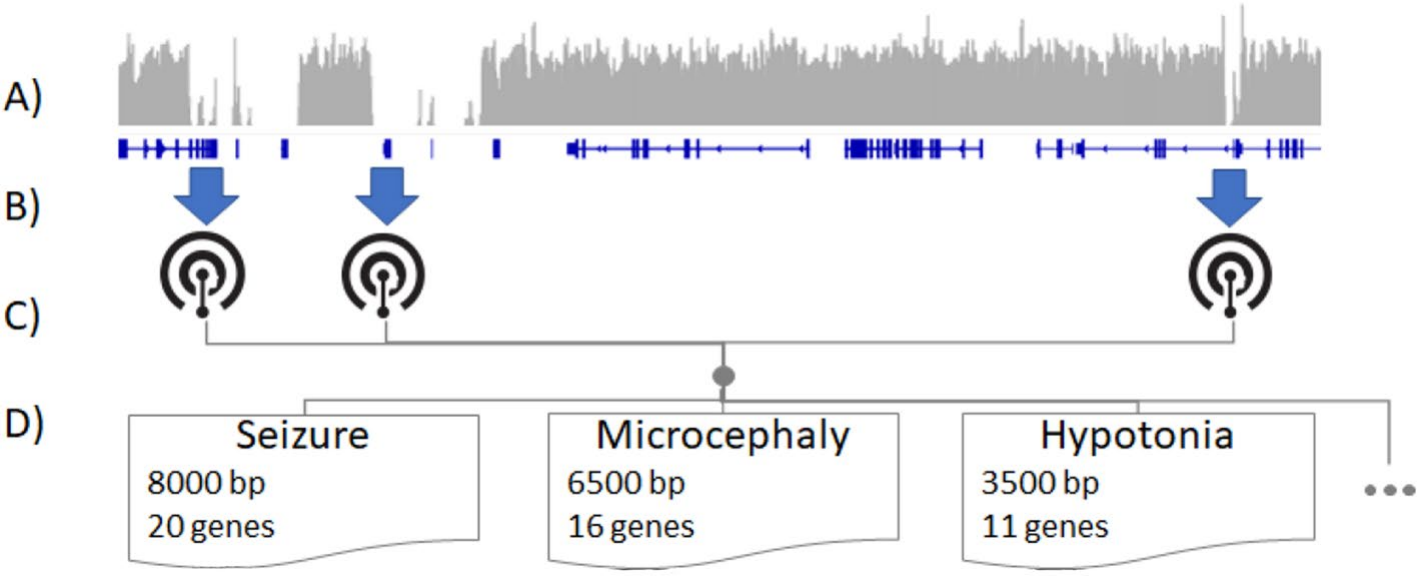
Illustrative schematic of determining HPO terms best assayable by LRS. (**A**) SRS genomic coverage (gray bars) averaged across hundreds of genomes is calculated for each gene (blue lines). (**B**) Disease genes are mapped to HPO terms. (**C**) Terms are assembled and (**D**) the number of genes and the SRS-uncoverable size are assembled for each HPO term. These can then be used to prioritize patients for long read sequencing.

In addition to the 22 nominated cases, we sought to identify whether certain phenotypes might especially benefit from LRS, particularly diseases for which the implicated gene may be hidden in an NGS dead zone. To do this, we determined the short read “dead zones” using 300 good-quality parental whole genome sequencing (WGS) samples (40× average coverage) that had been sequenced with short reads according to Rady Children’s Institute for Genomic Medicine (RCIGM) validated clinical protocol. Samples were evenly divided between sexes (150 female, 150 male) and came from unaffected parents. The dead zones were concordant with published results from Mandelker et al. who used WES on Illumina HiSeq 2500 platform, and were necessarily updated for the WGS on Illumina NovaSeq 6000 platform^9^. This adaptation between the technologies is necessary because NovaSeq technology uses an alternate, more rapid 2-channel sequencing by synthesis variation that could potentially have different boundaries for dead zones. We then determined the total number of coding bases for each gene in these dead zones. Following that, we associated the genes with their corresponding Human Phenotype Ontology (HPO) disease phenotypes, when applicable, as some of the genes do not yet have established relationships to human disease. Subsequently, we aggregated the dead zone bases across all genes for each phenotype, which allowed us to rank the phenotypes by the total size of associated dead zones (Fig. 2). After this analysis, a few genes, *TTN*, *SMN1/2* and *NEB*, tended to dominate HPO ranks due to the size of the gene and were removed from further analysis. The highest-ranked HPO term was “autosomal recessive inheritance”, followed by several HPO terms associated with male infertility, hearing impairment, and immune-mediated phenotypes (Supplementary Table 3). Our pediatric cohort lacked samples relating to the first two phenotypes, thus we chose to focus on immune-mediated phenotypes and thus selected four undiagnosed SRS cases from our biorepository with immunodeficiency as their presenting phenotype to receive LRS.

### SRS dead zones are uncovered by LRS

Whole genome sequencing by long reads successfully covered 98% of the total length of all NGS dead zones, allowing variants to be unambiguously called in these regions. The remaining 2% uncallable region was in one contiguous range of 180 kb (chr16:33,148,000–33,328,000). LRS identified an average of 54,900 (1.3%) additional small variants genome-wide, of which 440 (1.8%) were in near-coding regions and of these, 73% were in SRS dead zones or regions of low mapability^23^ the remainder were either large insertions or deletions that are difficult for short reads to map. Despite covering 98% of the genome and the methodological advantages of LRS, we were unable to find any molecular diagnoses neither in the 22 clinician-nominated cases nor their four affected family members. However, of the four immunodeficiency phenotype cases that underwent LRS, a maternally-inherited stop-loss variant (p.X420Yext27; VCV00011450) in *IKBKG* (inhibitor of nuclear factor kappa-b kinase, regulatory subunit gamma) was identified in one proband. This variant has been previously reported in ClinVar as Likely Pathogenic (LP). The variant is heterozygous in the mother and hemizygous in the male proband, who had been admitted to the neonatal intensive care unit (NICU) at 1 week of age. His physical exam on admission was notable for hepatosplenomegaly. Laboratory evaluation indicated abnormal immunoglobulins and imaging was remarkable for abnormal osseous mineralization. Clinical suspicion for an underlying genetic disorder, specifically a primary immunodeficiency, was high, but ultra-rapid WGS by SRS had failed to identify any pathogenic or likely pathogenic variants. The patient died in infancy from overwhelming infection. Post-mortem LRS identified the aforementioned stop-loss variant in *IKBKG*, a gene concealed in an NGS dead zone due to a pseudogene with greater than 99% homology. The c.1260 G > C substitution found in this case results in a loss of the stop codon at position 420 and changes this codon to a tyrosine, thereby creating a new stop codon at position 27 of the new reading frame (see Supplementary Fig. 2). *IKBKG* (inhibitor of nuclear factor kappa-b kinase, regulatory subunit gamma), previously referred to as *NEMO* (NF-kappa-b essential modulator), is essential to activation of the NF-κB pathway, a critical component of the host immune response to invading pathogens. Disease-causing variants in *IKBKG* have been associated with three clinical phenotypes: ectodermal dysplasia and immunodeficiency 1 [MIM:300291], immunodeficiency 33 [MIM:300636], and incontinentia pigmenti [MIM:308300], and depending on the functional impact of the sequence alteration, a wide phenotypic spectrum is possible. Hypomorphic hemizygous *IKBKG* disease-causing variants are typically associated with immunodeficiency with or without anhidrotic ectodermal dysplasia. Broadly speaking, disease-causing variants that alter the C-terminus zinc finger domain, as is the case for the variant in our proband, have a more severe clinical course. Previously, a male infant with a nearly identical *IKBKG* variant (c.1259A > G), resulting in loss of the stop codon (p. X420W) and the addition of 27 amino acids to the C terminus, was described with phenotypic overlap to our patient. He suffered from an immune deficiency manifested by recurrent infections, as well as osteopetrosis, and died from tuberculosis at age 2.5 years^24^. Another male was reported with an identical variant, identified by long-range PCR followed by Sanger sequencing, and a clinical presentation marked by osteopetrosis and recurrent infections with gram negative and positive bacteria, fungi, and mycobacteria, to which he ultimately succumbed at the age of 1.5 years^25^.

### Force calling in NGS dead zones to recover variants with low mapping scores

Visual inspection of the p.X420Yext27 variant in F12 revealed that the variant is also present in short-read sequencing as an apparent heterozygous mutation, however, because the reads overlapping the variant cannot be aligned to *IKBKG* or its pseudogene unambiguously, the reads were considered to be poor quality and no variant was called through standard pipelines. This is in contrast to long read sequencing which can align to this region unambiguously, and thus standard variant callers can identify variants with no modification.

To attempt to recover variants with low mapping scores such as in this case, we force called variants in NGS dead zones across 1226 samples. This was achieved by allowing all mapped reads, despite their quality, to be used for variant calling. We identified an average of 120.9 coding variants in dead zones per genome. We identified two additional variants in *IKBKG*: the first, p.X420Yext27, in a male infant (K1) with lethargy, hypotonia, oligohydramnios, hypoglycemia, progressive encephalopathy, and respiratory failure, was an identical variant to the variant seen in F12 and was apparently maternally inherited. The second was a novel variant (p.H413Y; VCV00068233) in another infant male (K2) with no parental NGS data available. Orthogonal interrogation of p.X420Yext27 in K1 failed to confirm the variant occurred in the coding sequence of *IKBKG* and thus was considered a false positive. The second variant was verified in both K2 and his mother. Though it was ultimately classified as a variant of uncertain significance (VUS) per ACMG criteria, there was credible phenotypic overlap. The proband was a two-week-old term male admitted to the pediatric intensive care unit (PICU) following an out-of-hospital cardiac arrest. His blood culture grew multiple gram-negative rods (*Enterobacter cloacae* and *Klebsiella pneumoniae*) and his cerebrospinal fluid also grew *Enterobacter cloacae*. The clinical genetics team was consulted and had concern for an underlying immunological defect. The medical geneticist recommended genome-wide sequencing to evaluate for an inborn error of immunity given the overwhelming infection in a previously healthy full-term infant. Despite maximal medical care with 3 vasoactive infusions, the patient died of refractory septic shock less than 3 days after admission.

## Discussion

Although the advent of whole genome sequencing in the past decade both significantly improved molecular diagnosis of genetic disorders and also substantially shortened historically step-wise, lengthy diagnostic odysseys, diagnostic rates have only modestly increased in the time since its debut, which is a challenge and source of frustration for clinicians and clinical laboratories alike. The present stagnation at an ~ 35% diagnostic rate is likely multi-factorial: fundamentally, the disease being evaluated may not be genetic in origin, the identified variant may not be interpretable based on current collective knowledge, or finally, the genetic insult may not be detectable by the interrogation method chosen (e.g. short read sequencing). Idiopathic, syndromic, severe, and early-onset diseases are generally suspected to be genetic in origin. Functional genomic approaches (e.g. RNAseq) may aid in evaluation of whether a variant (particularly non-coding variants) could be pathogenic, but this method can be hampered by uncertainty regarding which tissue(s) to sequence, availability of the desired tissue, and ambiguity surrounding the optimal time during development to sequence the tissue in the case of a congenital disease. Here, we chose to evaluate the ability of long-read sequencing to identify variants that may not be discoverable by short-read sequencing.

Of the five control cases, LRS was able to correctly identify the causative mutation in all. In one case, for which we had suspected a translocation by SRS, LRS revealed there was instead a pseudogene insertion that mimicked a translocation. Furthermore, we were also able to observe a difference in methylation by singe-molecule LRS in a case with uniparental disomy (UPD) of chromosome 15.

Despite extensive evaluation and an available pool of more than 1000 unsolved cases, none of the 22 clinician-nominated cases were solved with LRS (see Supplementary Table 4). This suggested that we consider a more focused application of LRS to select cases rather than indiscriminate use for all SRS-negative cases. Thus, we separately prioritized phenotypes for LRS by evaluating NGS dead zones associated with disease genes and their associated HPO terms. We then used these overrepresented phenotypes to select a subset of patients thought likely to benefit from LRS, specifically an undiagnosed group of four patients with immunodeficiency phenotypes. Of the four patients we selected for LRS, one (25%) received a molecular diagnosis: *IKBKG*-related immunodeficiency, lending support to this approach. We then sought to evaluate whether we could force-call variants in NGS dead zones to identify diagnostic variants using only short reads. We identified a known likely pathogenic variant and a VUS in *IKBKG* in two different patients (K1, K2) using this SRS force-calling approach. Interestingly, the LP mutation in K1 was a false positive call and likely occurs on the pseudogene, however, this was only determined by orthogonal confirmation (long-range, nested PCR). The *IKBKG* VUS in K2 had credible phenotypic overlap for a deceased neonatal patient. Overall LRS was able to detect pathogenic variants in two samples that would not normally be detectable by SRS (C4 and F12; Supplementary Table 4) and identified a false positive structural variant in one case. Although special processing can be used to detect variants in SRS dead zones, this can lead to false positives that perfectly mimic pathogenic variants. Thus, although this study was not designed to identify the false positive rate of SRS it is known that false positives for SVs is high^26^ and that force calling can induce additional false positives that can be correctly evaluated by LRS.

Our data support applying LRS with a more focused approach, such as identifying phenotypes that are more likely to benefit from LRS due to the coverage of NGS dead zones. However, LRS does have several methodologic advantages over SRS. First, the long-read sequencing presented here has high fidelity for small mutations and can identify nearly identical variants to those called by SRS; in the 35 samples that we analyzed, LRS identified an average of 1.3% additional small variants across the genome compared to SRS. Second, LRS performance is also superior to SRS for its ability to identify structural variants, particularly those mediated by repetitive or low-complexity elements. Third, single-molecule LRS can also detect genomic methylation defects and thus may serve as both a diagnostic and functional genomic approach. Fourth, LRS can detect variants in NGS dead zones that are not interrogatable by SRS. Additionally, for cases where individuals are adopted or biological parents are not available for other reasons, solo LRS has the ability to phase potential variants to determine, for example, if two suspicious variants are biallelic or not. Although SRS is extremely powerful, squeezing the full benefit from it requires fairly complex bioinformatics methods, typically the application of multiple approaches in tandem, and extensive databases for elimination of false positive discoveries. Even with these tools in place, numerous orthogonal approaches must be employed to confirm a suspicious variant and these may be expensive, time-consuming, and beyond the capabilities of the testing laboratory, thus requiring send-out. LRS as a first-pass approach may be especially beneficial for labs that do not have these informatic or wet-lab tools fully developed. By eliminating many false positives, the overall variant interpretation workflow is simplified. Furthermore, by evaluating both NGS dead zones and methylation patterns, the total number of potential additional tests needed to definitively conclude a patient does not have a molecular diagnosis (to the best of our present collective knowledge) is reduced, saving both time and effort on the part of the laboratory, informaticist, and clinician. The major disadvantages of LRS as compared to SRS are its higher cost, although this is likely to decrease in the future as the technology becomes more established. Future studies applying LRS to larger cohorts will be needed to determine the potential incremental diagnostic yield, effect on time to diagnosis, and which phenotypes or categories of patients would be best served by this new methodological approach.

## Methods

Informed and signed consent forms were obtained for all sequenced individuals. The project was approved by the Institutional Review Board (IRB) of the University of California at San Diego under protocol #160468 and WCG IRB under protocol #20171726 and was carried out in accordance with institutional guidelines and protocols. The project received non-significant risk status in a pre-Investigational Device Exemption submission to the Food and Drug Administration.

### Long-read sequencing

High-molecular weight DNA for each sample was sheared to a target size of 15–20 kb with a Diagenode Megaruptor three system (speed 31 then 32). Libraries were prepared with SMRTbell Express Template Prep Kit 2.0 (PacBio 100-938-900) following the manufacturer’s instructions (PacBio protocol 101-853-100). Libraries were selected for fragments longer than 10 kb using Sage Science PippinHT system with the “6–10 kb High Pass Marker 75 E” cassette definition.

Libraries were sequenced on the PacBio Sequel II and IIe Systems for 30 h movies with Sequel II Binding Kit 2.2 (PacBio 102-089-000) and Sequel II Sequencing Kit 2.0 (PacBio 101-826-100). HiFi reads were generated with CCS v6.0.0 with “-hifi-kinetics” (https://github.com/PacificBiosciences/ccs). The probability of methylation at CpG sites in reads was calculated with primrose v1.1.0 (https://github.com/PacificBiosciences/primrose) with default parameters.

HiFi reads were aligned to GRCh38 (GCA_000001405.15) using pbmm2 v1.4.0 (https://github.com/PacificBiosciences/pbmm2). Small variants were called with DeepVariant v1.1.0 using a two-pass workflow: preliminary variant calling, read phasing using WhatsHap 1.0, and final variant calling with phased reads. Structural variants were called with pbsv v2.6.0 (https://github.com/PacificBiosciences/pbsv) with default parameters for the discover step and “-A3 -O3” for the call step. De novo assemblies were generated with hifiasm v0.9 with default parameters^27–29^.

### Short-read sequencing

0.5–1 mL of blood was collected in an EDTA tube. Genomic DNA was isolated with an EZ1 Advanced XL robot and the EZ1 DSP DNA Blood Kit (Qiagen). DNA quality was assessed with the Quant-iT Picogreen dsDNA Assay Kit (Thermo Fisher Scientific) with the Gemini EM Microplate Reader (Molecular Devices). Genomic DNA was fragmented by sonication (Covaris), and bar-coded, paired-end, polymerase chain reaction (PCR)-free libraries were prepared for rWGS with TruSeq DNA LT kits (Illumina). Short-read clinical WGS was performed as previously described^17,30^ and in accordance with the best practices for the interpretation and reporting of clinical whole genome sequencing^31^: genomic DNA was fragmented by sonication and bar-coded, paired-end, PCR-free libraries were prepared for rWGS with TruSeq DNA LT kits (Illumina) or Hyper kits (KAPA Biosystems). Short-read sequences were aligned to human genome assemblies GRCh37.p13 (GCA_000001405.14) and GRCh38 (GCA_000001405.15) and variants were identified with the Illumina DRAGEN Bio-IT Platform (v.2.1.5 and v.3.4.5, Illumina. Structural variants were identified with Manta and CNVnator and/or DRAGEN-CNV, a combination that provided the highest sensitivity and precision. Structural variants were filtered to retain those affecting coding regions of known disease-associated genes and with allele frequencies < 2% in the RCIGM database. Nucleotide and structural variants were annotated, analyzed, and interpreted by clinical molecular geneticists^17^. Variant analysis was focused on variants in genes with a known relationship to human disease as previously described^17^. The differences with LRS results are not arising from the pipeline, but are intrinsic for the technology due to addressability of short reads to nearly identical reference genome segments^9^. Hard-to-call variants in such regions were force called using Platypus^32^.

## Supplementary Information

Supplementary Table 1.

## Data Availability

All reported genomic variants have been submitted to ClinVar (https://www.ncbi.nlm.nih.gov/clinvar/). ClinVar accession numbers: VCV00011450, VCV000429392, VCV00068233. Methylation data is available from https://platform.dnanexus.com/panx/projects/GFZ3p700Fv3GkZjX2PxK7xPj/data/Supplementary_data. Due to the vulnerable nature of working on pediatric patients with severe disease we are not allowed to share full BAMs publicly. We have made the regions of interest of our control samples freely available. Data can be made available to others by request. These PacBio read data are shared in the public DNAnexus project called “Approaches to long-read sequencing in a clinical setting to improve diagnostic rate:/Supplementary_data” and/or these downloadable links (each file ~ 3–12 Mb in size) https://dl.dnanex.us/F/D/Y1y58Gg07yQZP5J5pJ3kQPyKXYjj8FK1JVj8zj2v/C1.targetRegions.GRCh38.deepvariant.haplotagged.hifi.bam, https://dl.dnanex.us/F/D/fv9jZBg7YGK2Pbf81GPxjZPvv8xQ08gyxgjV4j96/C2.targetRegions.GRCh38.deepvariant.haplotagged.hifi.bam, https://dl.dnanex.us/F/D/gpVGjP8X4kv2Vpgx8KK5Pj6JbyQK60zgXJ03YJ6v/C3_lowDNA.targetRegions.GRCh38.deepvariant.haplotagged.hifi.bam, https://dl.dnanex.us/F/D/Z8vJp19B0GBy64jvqF9Fx36pFX8ZJ6xfxPjzp5by/C4.targetRegions.GRCh38.deepvariant.haplotagged.hifi.bam, https://dl.dnanex.us/F/D/65B9v7z56vYqPXvFxyBK4fBkvX574bZqfB7y9Gbk/C5.targetRegions.GRCh38.deepvariant.haplotagged.hifi.bam.
