# Supplementary material for: Approaches to long-read sequencing in a clinical setting to improve diagnostic rate
Source: Sci Rep. 2022 Oct 9;12:16945. doi: 10.1038/s41598-022-20113-x (PMC9548499; doi:10.1038/s41598-022-20113-x)
Supplement: Supplementary file 1 — Supplementary Table 1. [file 41598_2022_20113_MOESM1_ESM.pdf]

Supplemental Table 1. Unsolved cases and their clinical presentation

Family ID	Clinical Presentation
F1	Hypoplastic left heart syndrome, Atrial dysrhythmias, Chronic respiratory insufficiency and tachypnea, Bell shaped chest, Small embolic stroke, Left vocal cord paresis
F2	Siblings: Dystonia, Spastic paraparesis, Gross motor delay, Axonal sensory neuropathy
F3	Immunodeficiency, chronic lung disease, bronchiolitis obliterans
F4	Sunflower epilepsy, Developmental delay
F5	Skeletal dysplasia, Thrombocytopenia
F6	Siblings: Paroxysmal dyskinetic events, Gait ataxia
F7	Aseptic meningitis, Pulmonary infiltrates with respiratory distress, respiratory failure requiring mechanical ventilation, Numerous bony lesions involving bilateral humerus, clavicles, ribs, spine, and pelvis, and splenic involvement
F8	Mother and daughter, Hereditary neurodegenerative disorder, spasticity, sensory neuropathy
F9	Left hemihypertrophy, Postnatal overgrowth, Unilateral mesoaxial polydactyly of the left foot, Preauricular tag
F10	Heart failure, Hypotonia, Optic nerve hypoplasia
F11	Global developmental delay, Abnormality of movement, EEG abnormality, Hypertonia, Persistent head lag, Small for gestational age, Lactic acidosis
F12	Hepatosplenomegaly, Abnormality of the immune system, Bone mineral abnormality
F13	Autism Spectrum Disorder, Obesity- BMI 95th percentile, Global developmental delay, Hypotonia
F14	Siblings with Seizures, Abnormal bleeding, Encephalitis, Abnormality of the immune system, Maculopapular exanthema
F15	Influenza A encephalitis, Rhabdomyolysis, Respiratory failure, Seizures, Hyponatremia
F16	Brain anomalies identified at the anatomic scan at approx 20 weeks GA. Severe ventriculomegaly, midline fusion of the fornices, possible subependymal heterotopia, absent cavum septum pellucidum on fetal MRI
F17	Micrognathia, Low set ears, Redundant skin folds, Plagiocephaly,
F18	Cardiac arrest, Channelopathy family history
F19	Neuromyelitis optica, concern for underlying immune dysregulation
F20	Prematurity, Neonatal Heart failure
F21	Left ventricular dysfunction, Cardiac arrest, Cardiomegaly, Right ventricular hypertrophy, Prolonged QT interval, Respiratory distress, Neonatal hyperbilirubinemia, Edema
F22	Mixed ataxic and spastic palsy, Cerebellar atrophy
F23	Sensorineural Hearing Loss, Developmental delay, Acquired microcephaly, Abnormal interictal EEG, Multi-focal epilepsy and full body dystonia
F24	Cardiac arrest in infancy
F25	Autism spectrum features, bilateral idiopathic cerebellar strokes, bicuspid aortic valve
F26	Neonatal hemochromatosis, cholestasis

F2, F6, F8, F14 represent two members of the same family with the same phenotypic presentation. EEG = electroencephalogram, BMI = body mass index, GA = gestational age, MRI = magnetic resonance imaging

Supplemental Table 2. Recall of LRS and SRS for small variants (<50bp) across 32 samples.

Sample	%LRS recall (SNVs only)	%LRS recall (indels only)	%SRS recall (SNVs only)	%SRS recall (indels only)	Notes
C1	99.62%	97.60%	99.18%	97.58%	
C2	99.62%	94.64%	99.19%	94.56%	
C4	99.63%	97.22%	99.22%	97.05%	
C5	99.64%	97.62%	99.23%	97.48%	
F1	99.64%	97.84%	99.25%	97.77%	
F2	99.61%	97.63%	99.24%	97.53%	
F3	99.63%	95.83%	99.18%	95.88%	
F4	99.65%	97.65%	99.32%	97.82%	
F5	99.61%	97.72%	99.18%	97.72%	
F6	99.42%	95.85%	99.19%	96.30%	
F6.2	99.19%	93.08%	99.28%	93.94%	sibling in Family 6
F7	99.63%	96.02%	99.37%	96.51%	
F8	99.65%	97.90%	99.22%	97.82%	
F8.2	99.63%	97.72%	99.20%	97.67%	sibling in Family 8
F9	99.39%	96.40%	99.16%	97.30%	
F10	99.56%	97.44%	99.04%	97.54%	
F11	99.60%	97.76%	99.14%	97.63%	
F12	99.65%	97.48%	99.24%	97.39%	
F13	99.57%	97.07%	99.25%	97.59%	
F14	99.65%	97.95%	99.20%	97.84%	
F15	99.63%	97.57%	99.26%	97.69%	
F16	99.65%	97.54%	99.31%	97.74%	
F17	99.59%	95.95%	99.16%	95.86%	
F18	99.62%	95.83%	99.23%	95.97%	
F19	99.62%	96.95%	99.23%	97.02%	
F20	99.61%	97.62%	99.23%	97.89%	
F21	99.63%	97.62%	99.23%	97.63%	
F22	99.62%	97.28%	99.21%	97.10%	
F23	99.63%	97.75%	99.19%	97.43%	
F24	99.63%	94.89%	99.23%	94.76%	
F25	99.62%	97.75%	99.17%	97.84%	
F26	99.65%	98.05%	99.22%	97.77%	
**Mean (Std.dev)**	**99.60% (0.09%)**	**96.98% (1.16%)**	**99.22% (0.06%)**	**97.05% (1.05%)**	

Notes:

* sample C3 had very degraded DNA library (no redraw possible), leading to uncharacteristic bad performance, and not used for this comparison
* confident regions of HG38 reference genome are fixed to those of HG002 GIAB reference variant set (per best practices of variant assessment [X3])

Supplemental Table 3. Top 35 HPO terms and their associated genes and dead zone sizes.

HPO term	Number of Genes	Dead Zone Size (bp)	Genes
Autosomal recessive inheritance	20	35586	*ACAN C4A HERC2 ADAMTSL2 PHC1 FLG GRAP CORO1A USP18 PMS2 HYDIN STRC MSTO1 NCF1 PIEZO2 OCLN STAT5B OTOA TNXB ANAPC1*
Azoospermia	12	28532	*PRY2 PRY BPY2 VCY CDY1 CDY2A DAZ1 HSFY1 STRC RBMY1A1 DAZ3 DAZ2*
Y-linked inheritance	11	25506	*PRY2 PRY BPY2 VCY CDY1 CDY2A DAZ1 HSFY1 RBMY1A1 DAZ3 DAZ2*
Autosomal dominant inheritance	19	25001	*ACAN HERC2 CEL CES1 CHRNA7 FCGR2B FLG TUBB2B KRT81 KRT86 PMS2 MSTO1 RPS17 PIEZO2 STAT5B CFC1 TNXB TUBB2A ZP3*
Male infertility	7	22308	*DAZ1 HYDIN STRC RBMY1A1 DAZ3 DAZ2 DAZ4*
Oligospermia	6	21117	*DAZ1 STRC RBMY1A1 DAZ3 DAZ2 DAZ4*
Cryptorchidism	10	19495	*HERC2 DAZ1 GTF2I RBMY1A1 NCF1 PIEZO2 ANAPC1 DAZ3 DAZ2 DAZ4*
Non-obstructive azoospermia	5	18091	*DAZ1 RBMY1A1 DAZ3 DAZ2 DAZ4*
Decreased testicular size	5	18091	*DAZ1 RBMY1A1 DAZ3 DAZ2 DAZ4*
Sensorineural hearing impairment	6	16121	*GRAP GTF2I DUX4 STRC NCF1 OTOA*
Meningitis	3	16042	*C4A C4B NCF1*
Skeletal muscle atrophy	4	14395	*DUX4 TUBB2B PIEZO2 TNXB*
Seizure	12	14366	*IKBKG C4A HERC2 CHRNA7 ADAMTSL2 FCGR2B USP18 TUBB2B PMS2 PIEZO2 OCLN TUBB2A*
Keratosis pilaris	3	12957	*FLG KRT81 KRT86*
Short stature	15	12765	*ACAN IKBKG HERC2 CHRNA7 ADAMTSL2 PHC1 GTF2I TUBB2B MSTO1 NCF1 RPS17 PIEZO2 STAT5B SPIDR ANAPC1*
Abnormal eyelash morphology	3	12384	*DUX4 KRT81 KRT86*
Elevated circulating creatine kinase concentration	5	12344	*GTF2I DUX4 MSTO1 NCF1 PIEZO2*
Eczematoid dermatitis	2	12242	*FLG NCF1*
Hyperlordosis	3	12217	*GTF2I DUX4 NCF1*

Abnormality of cardiovascular system morphology	2	12074	*CHRNA7 DUX4*
Asthma	1	12048	*FLG*
Childhood onset	1	12048	*FLG*
Ichthyosis	1	12048	*FLG*
Dry skin	1	12048	*FLG*
Palmar hyperlinearity	1	12048	*FLG*
Cataract	8	11689	*IKBKG C4A GTF2I TUBB2B KRT81 KRT86 NCF1 OCLN*
Mask-like facies	2	11518	*DUX4 PIEZO2*
Palpebral edema	1	11475	*DUX4*
EMG abnormality	1	11475	*DUX4*
Abnormal retinal vascular morphology	1	11475	*DUX4*
Hyperreflexia	6	10557	*C4A PHC1 GTF2I TUBB2B NCF1 OCLN*
Arthralgia	4	10482	*C4A GTF2I NCF1 TNXB*
Gait disturbance	4	10140	*IKBKG C4A TUBB2B PMS2*
Gastrointestinal hemorrhage	4	10131	*C4A FCGR2C PMS2 TNXB*
Fatigue	4	10036	*C4A PMS2 STAT5B TNXB*

EMG = electromyography

Supplemental Table 4. Solved and unsolved cases by sequencing type

Sample	Detectable by SRS	Detectable by LRS
C1	Yes, as a copy gain	Yes, as a translocation
C2	False positive translocation	True negative translocation
C3	Yes	Yes
C4	No, except by special processing	Yes
C5	UPD but not methylation detectable by special processing	Yes
F1	undiagnosed	undiagnosed
F2	undiagnosed	undiagnosed
F3	undiagnosed	undiagnosed
F4	undiagnosed	undiagnosed

F5	undiagnosed	undiagnosed
F6	undiagnosed	undiagnosed
F7	undiagnosed	undiagnosed
F8	undiagnosed	undiagnosed
F9	undiagnosed	undiagnosed
F10	undiagnosed	undiagnosed
F11	undiagnosed	undiagnosed
F12	Yes, with special processing	Yes
F13	undiagnosed	undiagnosed
F14	undiagnosed	undiagnosed
F15	undiagnosed	undiagnosed
F16	undiagnosed	undiagnosed
F17	undiagnosed	undiagnosed
F18	undiagnosed	undiagnosed
F19	undiagnosed	undiagnosed
F20	undiagnosed	undiagnosed
F21	undiagnosed	undiagnosed
F22	undiagnosed	undiagnosed
F23	undiagnosed	undiagnosed
F24	undiagnosed	undiagnosed
F25	undiagnosed	undiagnosed
F26	undiagnosed	undiagnosed

Supplemental Figure 1. Long read results from positive control samples. A) Ribbon plot showing apparent translocation from chromosome 11 (orange) to chromosome 22 (green), projected onto assembled alternate contig (black) for control sample C1. B) IGV screen shot showing insertion (purple)

of ~3.5Kbp into SCAI in control sample C2. C) BLAT results from 3.5Kbp insertion (grey) shows alignment to processed pseudogene SMAD4. D) Ribbon plot showing inversion on chromosome 8 (purple) projected onto assembled alternate contig (black) for control sample C3. E) IGV screen shot showing 2bp deletion in *IKBKG* in sample C4.

A

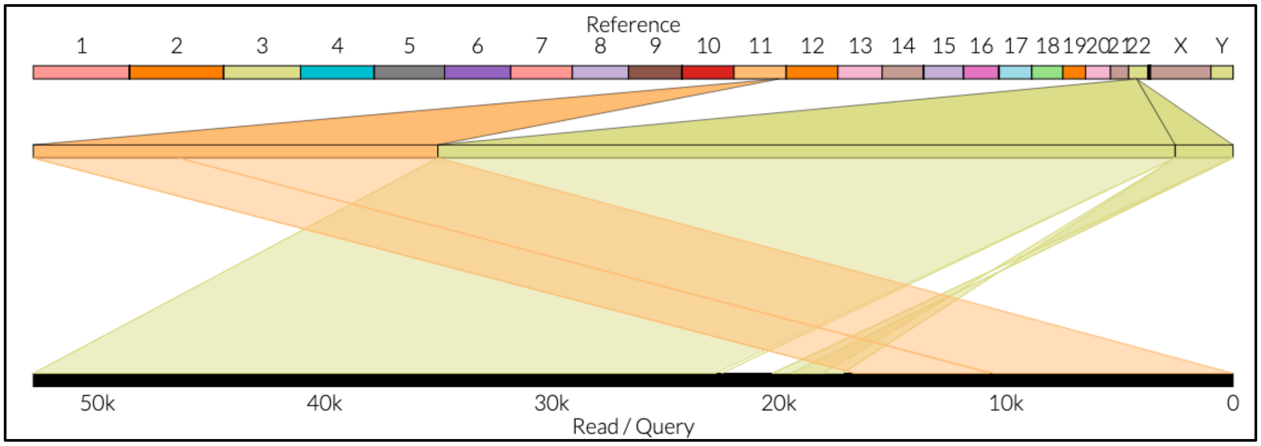

B

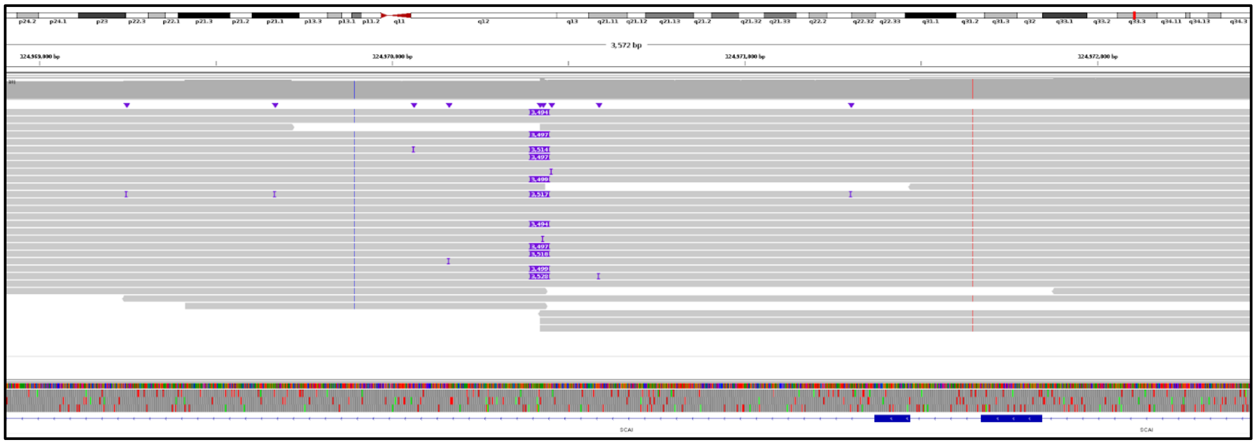

C

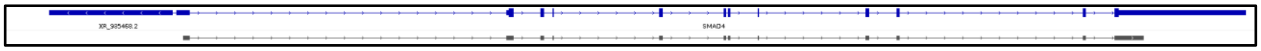

D

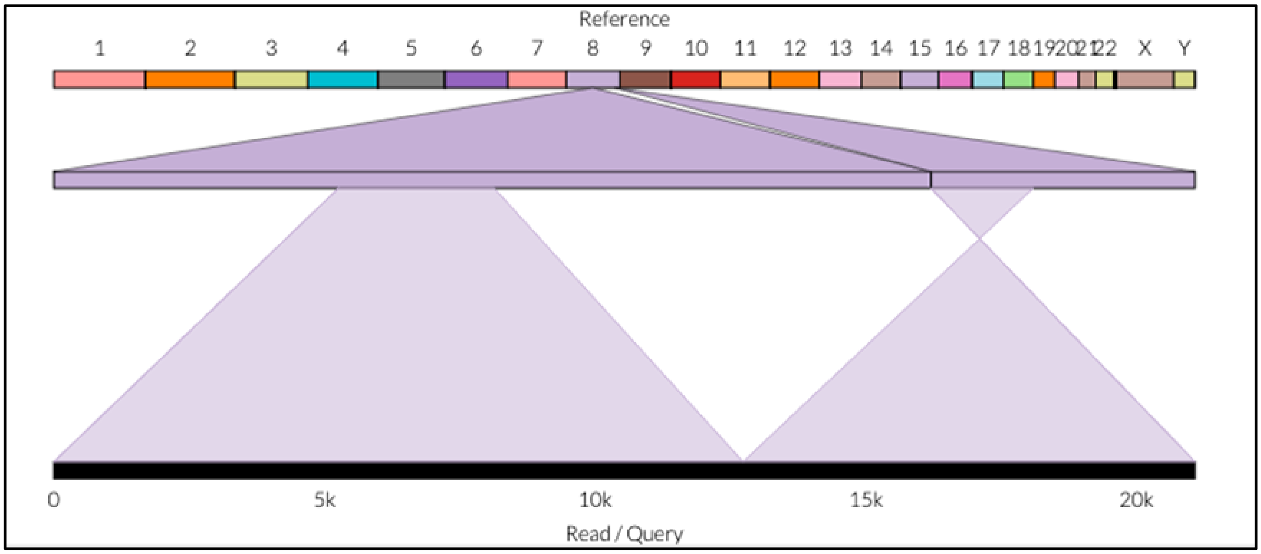
Reference
1
2
3
4
5
6
7
8
9
10
11
12
13
14
15
16
17
18
19
20
21
22
X
Y
0
5k
10k
15k
20k
Read / Query

E

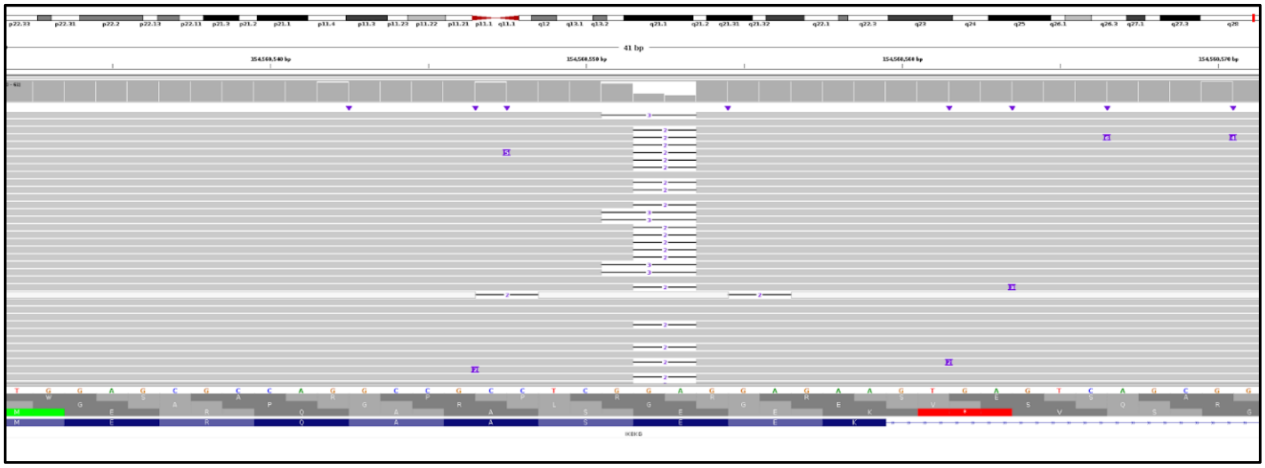

Supplemental Figure 2. Stop-loss variant in IKBKG causes an extension of the reading frame by 27 amino acids. Genomic DNA is shown with putative protein translation below. Alternating codons are underlined. Stop codon (TER) shown in bold. DNA variant and subsequent change to the amino acid residue shown in bold red.

TGCATTGAGTAGGGCCGGCCAGTGCAAGGCCACTGCCTGCCGAGGACGTGCCCGGGACCGTGCAGTCTGCGCTTTCCTCTCCCGCCTGCCTAG

C I E **TER**

TGCATTGAGTA**C**GGCCGGCCAGTGCAAGGCCACTGCCTGCCGAGGACGTGCCCGGGACCGTGCAGTCTGCGCTTTCCTCTCCCGCCTGCCTAG

C I E **Y** G R P V Q G H C L P R T C P G P C S L R F P L P P A **TER**
